# Supplementary material for: Burden changes in notifiable infectious diseases in Taiwan during the COVID-19 pandemic
Source: PeerJ. 2024 Sep 9;12:e18048. doi: 10.7717/peerj.18048 (PMC11391939; doi:10.7717/peerj.18048)
Supplement: Supplemental Information 1 [file peerj-12-18048-s001.docx]

**Supplementary Information**

**Additional fiel 1: Figure S1. Share of total disease burden by casuse, Taiwan, 2005 to 2009.**

**Additional file 2: Figure S2.** Flow chart of inclusion of disease notification categories.

**Additional file 3: Figure** **S3.** TreeMap diagrams displays the relative contribution percentage for specific disease notification categories. Six diagrams represent the disease burden of years of life lost (YLL), years lost due to disability (YLD), and disability-adjusted life years (DALYs) in specific 2010 and 2020 year.

**Additional file 3: Table S1.** National surveillance of infectious diseases in Taiwan: relative change (%) in number of cases and deaths in 2010, compared to the data in 2020. The formal category “Severe Pneumonia with Novel Pathogens” refers to COVID-19.

**Additional file 4: Table S2.** The codes translated from ICD-9-CM to ICD-10-CM in Taiwan.

**Additional file 5: Table S3.** 2010 and 2020 life-table with a life expectancy varied with the birth year.

**Additional file 6: Table S4.** Summary of data sources and DALY parameters for individual diseases notification categories - Airborne/droplet transmitted diseases.

**Additional file 7: Table S5.** Summary of data sources and DALY parameters for individual diseases notification categories - Fecal-oral transmitted diseases.

**Additional file 8: Table S6.** Summary of data sources and DALY parameters for individual diseases notification categories - Direct-contact and Vector-borne transmitted diseases.

**Additional file 9: Table S7.** Summary of data sources and DALY parameters for individual diseases notification categories - Sexually and blood-borne transmitted diseases.

**Appendix Fig 1.**


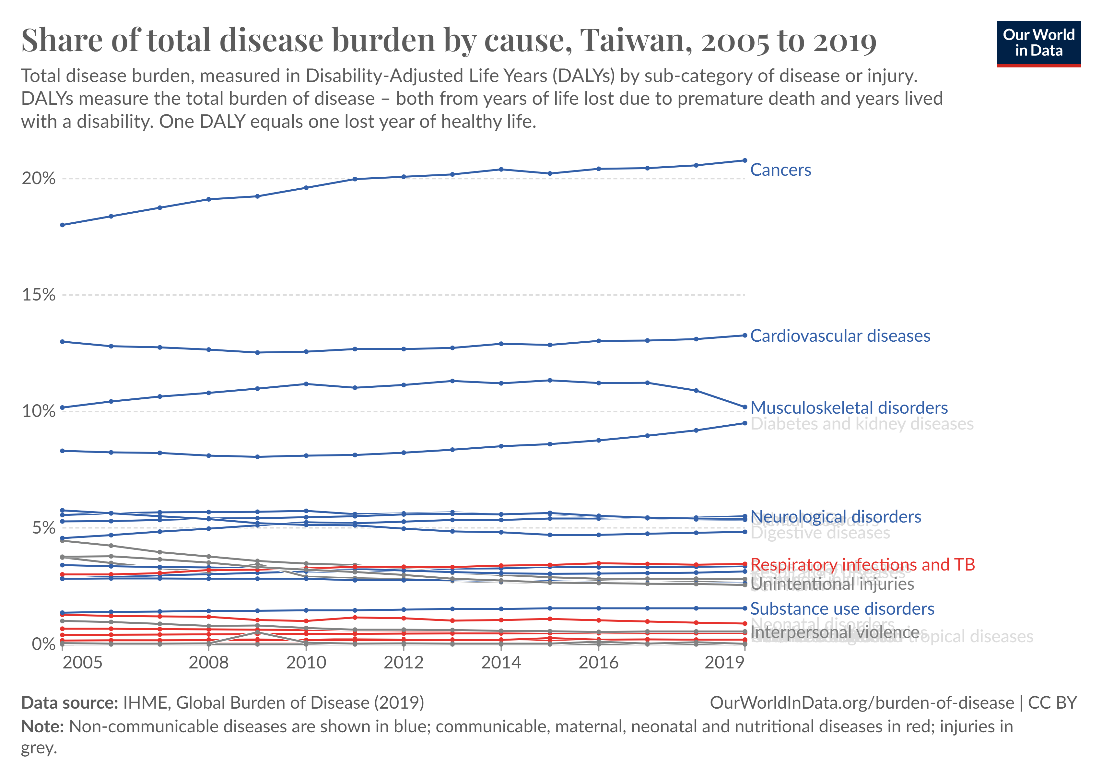


**Reference**

Institute for Health Metrics and Evaluation, Global Burden of Disease (2019) – processed by Our World in Data. “ HIV/AIDS and STIs” [dataset]. Institute for Health Metrics and Evaluation, Global Burden of Disease (2019)

**Appendix Fig 2.**

98 diseases notification categories under surveillance in Taiwan, according to the Taiwan Centers for Disease Control (CDC) in 2010 and 2020

74 diseases notification categories under national surveillance in Taiwan in 2010 and 2020

48 diseases notification categories under national surveillance in Taiwan in 2010 and 2020

43 diseases notification categories under national surveillance in Taiwan, in 2010 and 2020, included in this study.

24 categories excluded (The reporting cases = 0 cases both in 2010 and 2020)

26 categories excluded (There is no surveillance data shown on the Statistics of Communicable Diseases and Surveillance Report)

2 categories excluded (Unusable to confirm the death cases because the different code between ICD-9 and ICD-10 classify)

3 categories excluded (Unavailable parameters, ex: disability weight, disability duration, and severity distribution)

**Appendix Fig 3.**

**2010**

**2020**

**YLLs**

**YLDs**

**DALYs**


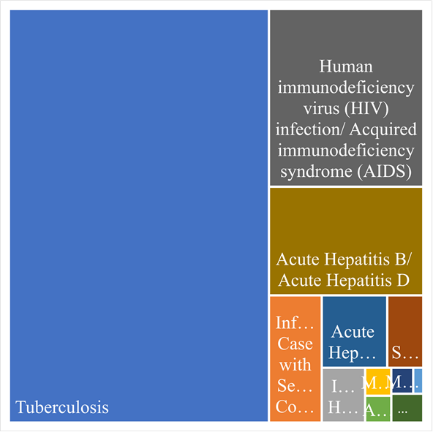

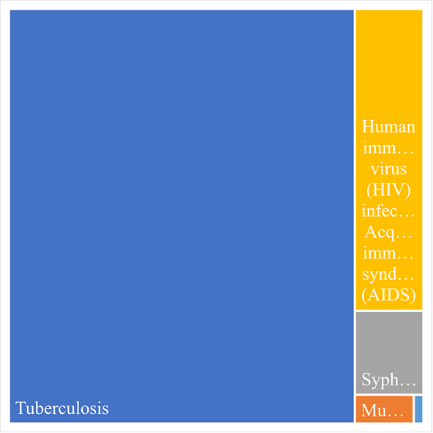

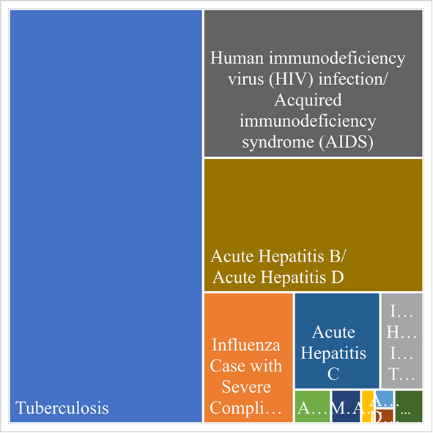

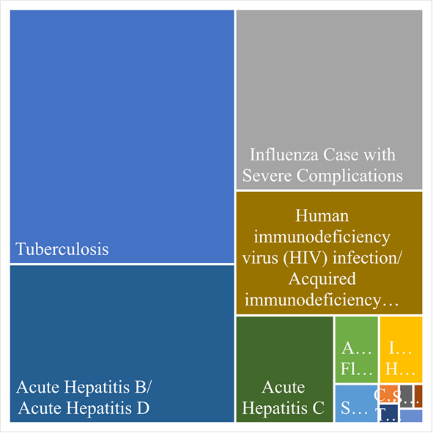

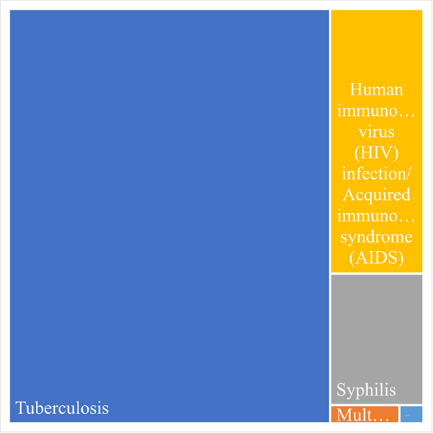

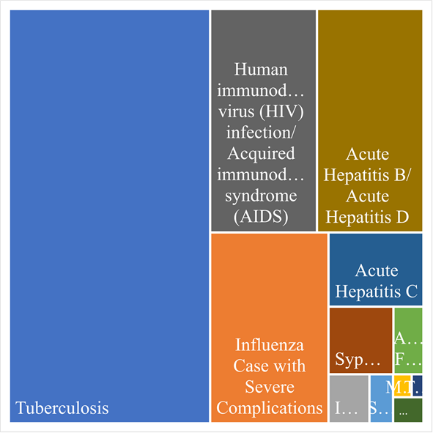


**Appendix Table 1** National surveillance of infectious diseases in Taiwan: relative change (%) in number of cases and deaths in 2010, compared to the data in 2020. The formal category “Severe Pneumonia with Novel Pathogens” refers to COVID-19.

| **Notifiable infectious disease category** |  |  | |  | **Change in case numbers by reporting age groups (yrs)** | | | | | | |  |  |  |
| --- | --- | --- | --- | --- | --- | --- | --- | --- | --- | --- | --- | --- | --- | --- |
|  | Number of case  Case | | | Change (%) | <1 | 1~4 | 5~14 | 15~24 | 25~39 | 40~64 | >=65 | Number of death | | Change (%) |
| **Airborne/droplet transmitted diseases** | 2010 | | 2020 |  |  |  |  |  |  |  |  | 2010 | 2020 |  |
| Tuberculosis | 13237 | | 7823 | -40.9 |  |  |  |  |  |  |  | 654 | 460 | -29.7 |
| Complicated Varicella | 9218 | | 54 | -99.4 |  |  |  |  |  |  |  | 2 | 1 | -50.0 |
| Mumps | 1125 | | 498 | -55.7 |  |  |  |  |  |  |  | 0 | 2 | NA |
| Pertussis | 61 | | 5 | -91.8 |  |  |  |  |  |  |  | 0 | 0 | NA |
| Influenza Case with Severe Complications | 882 | | 444 | -49.7 |  |  |  |  |  |  |  | 58 | 184 | 217.2 |
| Invasive Haemophilus Influenzae Type b Infection | 12 | | 3 | -75.0 |  |  |  |  |  |  |  | 22 | 15 | -31.8 |
| Legionnaires' Disease | 102 | | 326 | 219.6 |  |  |  |  |  |  |  | 0 | 0 | NA |
| Q fever | 89 | | 14 | -84.3 |  |  |  |  |  |  |  | 0 | 0 | NA |
| Multidrug-Resistant Tuberculosis | 156 | | 74 | -52.6 |  |  |  |  |  |  |  | 0 | 0 | NA |
| Measles | 12 | | 0 | -100.0 |  |  |  |  |  |  |  | 0 | 0 | NA |
| Rubella | 21 | | 0 | -100.0 |  |  |  |  |  |  |  | 0 | 0 | NA |
| Meningococcal Meningitis | 7 | | 6 | -14.3 |  |  |  |  |  |  |  | 0 | 0 | NA |
| Hantavirus Syndrome | 1 | | 11 | 1000.0 |  |  |  |  |  |  |  | 0 | 0 | NA |
| Severe Pneumonia with Novel Pathogens | 0 | | 823 | NA |  |  |  |  |  |  |  | 0 | 7 | NA |
| **Fecal-oral transmitted diseases** |  | |  |  |  |  |  |  |  |  |  |  |  |  |
| Amoebiasis | 262 | | 250 | -4.6 |  |  |  |  |  |  |  | 2 | 0 | -100.0 |
| Shigellosis | 172 | | 151 | -12.2 |  |  |  |  |  |  |  | 0 | 0 | NA |
| Acute Hepatitis A | 110 | | 74 | -32.7 |  |  |  |  |  |  | 220% | 1 | 0 | -100.0 |
| Acute Flaccid Paralysis and Poliomyelitis | 49 | | 33 | -32.7 |  |  |  |  |  |  |  | 6 | 13 | 116.7 |
| Botulinus | 11 | | 1 | -90.9 |  |  |  |  |  |  |  | 0 | 0 | NA |
| Typhoid Fever | 33 | | 10 | -69.7 |  |  |  |  |  |  |  | 0 | 0 | NA |
| Paratyphoid Fever | 12 | | 0 | -100.0 |  |  |  |  |  |  |  | 0 | 0 | NA |
| Toxoplasmosis | 5 | | 17 | 240.0 |  |  |  |  |  |  |  | 0 | 1 | NA |
| Cholera | 5 | | 1 | -80.0 |  |  |  |  |  |  |  | 1 | 0 | -100.0 |
| Acute Hepatitis E | 7 | | 7 | 0.0 |  |  |  |  |  |  |  | 0 | 0 | NA |
| Listeriosis | 0 | | 142 | NA |  |  |  |  |  |  |  | 0 | 1 | NA |
| **Direct-contact transmitted diseases** |  | |  |  |  |  |  |  |  |  |  |  |  |  |
| Leptospirosis | 77 | | 86 | 11.7 |  |  |  |  |  |  |  | 0 | 0 | NA |
| Melioidosis | 45 | | 20 | -55.6 |  |  |  |  |  |  |  | 5 | 0 | -100.0 |
| Tetanus | 12 | | 8 | -33.3 |  |  |  |  |  |  |  | 1 | 1 | 0.0 |
| Hansen’s Disease | 5 | | 7 | 40.0 |  |  |  |  |  |  |  | 3 | 0 | -100.0 |
| **Vector-borne transmitted diseases** |  | |  |  |  |  |  |  |  |  |  |  |  |  |
| Dengue Fever | 1917 | | 137 | -92.9 |  |  |  |  |  |  |  | 3 | 0 | -100.0 |
| Scrub Typhus | 402 | | 422 | 5.0 |  |  |  |  |  |  |  | 0 | 0 | NA |
| Japanese Encephalitis | 33 | | 21 | -36.4 |  |  |  |  |  |  |  | 0 | 0 | NA |
| Malaria | 21 | | 2 | -90.5 |  |  |  |  |  |  |  | 0 | 0 | NA |
| Lyme Disease | 0 | | 1 | NA |  |  |  |  |  |  |  | 0 | 0 | NA |
| Zika Virus Infection | 0 | | 2 | NA |  |  |  |  |  |  |  | 0 | 0 | NA |
| **Sexually transmitted and blood-borne diseases** |  | |  |  |  |  |  |  |  |  |  |  |  |  |
| Gonorrhea | 2265 | | 7082 | 212.7 |  |  |  |  |  |  |  | 2 | 3 | 50.0 |
| Syphilis | 6482 | | 8799 | 35.7 |  |  |  |  |  |  |  | 0 | 0 | NA |
| Human immunodeficiency virus (HIV) infection | 1796 | | 1390 | -22.6 |  |  |  |  |  |  |  | 115 | 80 | -30.4 |
| Acquired immunodeficiency syndrome (AIDS) | 1087 | | 800 | -26.4 |  |  |  |  |  |  |  |  |  |  |
| Acute Hepatitis B | 172 | | 108 | -37.2 |  |  |  |  |  |  |  | 165 | 198 | 20.0 |
| Acute Hepatitis C | 41 | | 602 | 1368.3 |  |  |  |  |  |  |  | 54 | 70 | 29.6 |
| Acute Hepatitis D | 1 | | 0 | -100.0 |  |  |  |  |  |  |  | 0 | 0 | NA |

| - Change (%) | | | | | |  |
| --- | --- | --- | --- | --- | --- | --- |
| 0-20 | 20-40 | 40-60 | 60-80 | 80-100 | 0% | No case |
|  |  |  |  |  |  |  |
|  |  |  |  |  |  |  |
| 0-20 | 20-40 | 40-60 | 60-80 | 80-100 | 100-200 | >200 |
| + Change (%) | | | | | | |

**Appendix Table 2** The codes translated from ICD-9-CM to ICD-10-CM in Taiwan.

| **Diseases** | **ICD-9-CM** | **ICD-10-CM** |
| --- | --- | --- |
| **Airborne/droplet transmitted diseases** |  |  |
| Tuberculosis | 010-018 | A15-, A17-, A18-, A19-, J65 |
| Complicated Varicella | 52 | B01- |
| Mumps | 072 | B26- |
| Pertussis | 033.0 | A37- |
| Influenza Case with Severe Complications | 487 | J10-, J11- |
| Invasive Haemophilus Influenzae Type b Infection | 320.0 | G00.0, A41.3, B96.3, J14 |
| Legionnaires' Disease | 482.84 | A48.1 |
| Q fever | 083.0 | A78 |
| Multidrug-Resistant Tuberculosis | 010-018 | Z16.342 |
| Measles | 055 | B05- |
| Rubella | 056 | B06- |
| Meningococcal Meningitis | 036.0 | A39- |
| Hantavirus Syndrome | 078.6, 480.8 | A98.5, B33.4 |
| Severe Pneumonia with Novel Pathogens | NA | U07.1, U07.2 |
| **Fecal-oral transmitted diseases** |  |  |
| Amoebiasis | 006 | A06- |
| Shigellosis | 004 | A03- |
| Acute Hepatitis A | 070.0, 070.1 | B15- |
| Acute Flaccid Paralysis and Poliomyelitis | 045 | A80, A80.0, A80.1, A80.2, A80.3, A80.30, A80.39, A80.9 |
| Botulinus | 005.1 | A05.1, A48.5- |
| Typhoid Fever | 002.0 | A01.0 |
| Paratyphoid Fever | 002.1 | A01.1, A01.2, A01.3, A01.4 |
| Toxoplasmosis | 130 | B58-, P37.1 |
| Cholera | 001 | A00- |
| Acute Hepatitis E | 070.43, 070.53 | B17.2 |
| Listeriosis | 027.0 | A32-, P37.2 |
| **Direct-contact transmitted diseases** |  |  |
| Leptospirosis | 100 | A27- |
| Melioidosis | 025 | A24.1, A24.2, A24.3, A24.9 |
| Tetanus | 037 | A34, A35 |
| Hansen’s Disease | 030 | A30- |
| **Vector-borne transmitted diseases** |  |  |
| Dengue Fever | 061, 065.4 | A90, A91 |
| Scrub Typhus | 081.2 | A75.3 |
| Japanese Encephalitis | 062.0 | A83.0 |
| Malaria | 084 | B50-, B51-, B52-, B53-, B54 |
| Lyme Disease | 088.81, 104.8 | A69.2- |
| Zika Virus Infection | 066.3 | A92.5 |
| **Sexually and blood-borne transmitted diseases** |  |  |
| Gonorrhea | 098 | A54-, O98.2 |
| Syphilis | 090-097 | A50-, A51-, A52-, A53-, O98.1- |
| Human immunodeficiency virus (HIV) infection | 042 | B20-, O98.7-, Z21 |
| Acquired immunodeficiency syndrome (AIDS) | 042 | B20-, O98.7- |
| Acute Hepatitis B | 070.20, 070.21, 070.30, 070.31 | B16- |
| Acute Hepatitis C | 070.41, 070.51 | B17.1- |
| Acute Hepatitis D | 070.42, 070.52 | B17.0, B16.0, B16.1 |

**Appendix Table 3** 2010 and 2020 life-table with a life expectancy varied with the birth year.

|  | Age groups | | | | | | |
| --- | --- | --- | --- | --- | --- | --- | --- |
| 2010 year | <1 | 1~4 | 5~14 | 15~24 | 25~39 | 40~64 | >=65 |
| All population | 78.25 | 76.14 | 69.23 | 59.41 | 47.4 | 29.51 | 12.14 |
| Male | 75.01 | 72.91 | 66 | 56.24 | 44.4 | 27.21 | 11.06 |
| Female | 82 | 79.88 | 72.96 | 63.07 | 50.87 | 32.06 | 13.22 |
| 2020 year | <1 | 1~4 | 5~14 | 15~24 | 25~39 | 40~64 | >=65 |
| All population | 80.01 | 77.86 | 70.92 | 61.1 | 49.01 | 30.88 | 13.17 |
| Male | 76.58 | 74.43 | 67.51 | 57.72 | 45.71 | 28.18 | 11.41 |
| Female | 83.93 | 81.77 | 74.83 | 64.95 | 52.75 | 33.86 | 14.54 |

Data source: Taiwan Ministry of the Interior. Abridged life table 2010 and 2020, 〈<https://www.moi.gov.tw/cl.aspx?n=3053>〉 [Accessed 15 January 2023] (Taiwan MOI, 2021 and 2020).

**Appendix Table 4** Summary of data sources and DALY parameters for individual diseases notification categories - Airborne/droplet transmitted diseases.

| **Notifiable infectious disease** | **Disease severity** | **Severity distribution (%)** | **Disability duration** | **Disability weights** | **Source** |
| --- | --- | --- | --- | --- | --- |
| Tuberculosis | - | - | 3 yr | 0.333 (0.224-0.454)^b^ | Abbafati et al. (2020) |
| Complicated Varicella | - | - | 7 d | 0.006 (0.002-0.012)^b^ |  |
| Mumps | Moderate | 32-40 | 0.019-0.027 yr | 0.051 (0.039-0.06)^a^ | Cassini et al. (2018) |
|  | Severe | 60-68 | 0.019-0.038 yr | 0.125 (0.104-0.152)^a^ |  |
| Pertussis | - | - | 50 d | 0.051 (0.032-0.074)^b^ | Abbafati et al. (2020) |
| Influenza Case with Severe Complications | Moderate | 85 | 7.79 d | 0.051 (0.032-0.074)^b^ |  |
|  | Severe | 15 |  | 0.133 (0.088-0.19)^b^ |  |
| Invasive Haemophilus Influenzae Type b Infection | - | - | 0.019 yr | 0.655 (0.579-0.727)^a^ | Cassini et al. (2018) |
| Legionnaires' Disease | Moderate | 26-31 | 0.022-0.036 yr | 0.051 (0.039-0.06)^a^ |  |
|  | Severe | 46.7-53.2 |  | 0.125 (0.104-0.152)^a^ |  |
|  | Intensive Care Unit | 20.7-22.2 |  | 0.655 (0.579-0.727)^a^ |  |
| Q fever | Mild | 95-98 | 0.038 yr | 0.007 (0.005-0.01)^a^ |  |
|  | Severe | 2-5 |  | 0.125 (0.104-0.152)^a^ |  |
| Multidrug-Resistant Tuberculosis | - | - | 3 yr | 0.333 (0.224-0.454)^b^ | Abbafati et al. (2020) |
| Measles | Moderate | 50 | 10 d | 0.051 (0.032-0.074)^b^ |  |
|  | Severe | 50 |  | 0.133 (0.088-0.19)^b^ |  |
| Rubella | - | - | 0.008 yr | 0.007 (0.005-0.01)^a^ | Cassini et al. (2018) |
| Meningococcal Meningitis | - | - | 4 weeks | 0.133 (0.088-0.19)^b^ | Abbafati et al. (2020) |
| Hantavirus Syndrome | - | - | 3-8 d | 0.472 (0.411-0.532)^b^ | Taiwan CDC (2022); Ock et al. (2019) |
| Severe Pneumonia with Novel Pathogens | Asymptomatic | 42 | 14 d | 0 | Rommel et al. (2021) |
|  | Mild | 25 | 14 d | 0.006 |  |
|  | Moderate | 27 | 14 d | 0.051 |  |
|  | Severe | 5 | 21 d | 0.133 |  |
|  | Critical | <1 | 32 d | 0.655 |  |

^a^ Mean (Range). ^b^ 95% CI

**Appendix Table 5** Summary of data sources and DALY parameters for individual diseases notification categories - Fecal-oral transmitted diseases.

| **Notifiable infectious disease** | **Disease severity** | **Severity distribution (%)** | **Duration** | **Disability weights** | **Source** |
| --- | --- | --- | --- | --- | --- |
| Amoebiasis | Mild | 91 | 7 d | 0.074 | Lai and Yu (2020) |
|  | Moderate | 8.5 |  | 0.188 |  |
|  | Severe | 0.5 |  | 0.247 |  |
| Shigellosis | Mild | 91 | 7 d | 0.074 |  |
|  | Moderate | 8.5 |  | 0.188 |  |
|  | Severe | 0.5 |  | 0.247 |  |
| Acute Hepatitis A | Moderate | 40 | 21 d | 0.051 (0.032-0.074)^b^ | Abbafati et al. (2020); Lai and Yu (2020) |
|  | Severe | 60 |  | 0.133 (0.088-0.19)^b^ |  |
| Acute Flaccid Paralysis and Poliomyelitis | Mild | 70.59 | 0.019 yr | 0.007 (0.005-0.01)^a^ | Cassini et al. (2018) |
|  | Moderate | 17.65 | 0.05-0.027 yr | 0.051 (0.039-0.06)^a^ |  |
|  | Severe | 11.76 | 0.011-0.038 yr | 0.125 (0104-0.152)^a^ |  |
| Botulinus | - | - | 7 d | 0.183 | Lai and Yu (2020) |
| Typhoid Fever | Moderate | 35.0 (26.0-44.3)^b^ | 14 d (7-21)^c^ | 0.051 (0.032-0.074)^b^ | Abbafati et al. (2020); Stanaway et al. (2019) |
|  | Severe | 47.75 (38.0-57.4)^b^ |  | 0.133 (0.088-0.19)^b^ |  |
|  | Gastrointestinal bleeding | 0.25 (0-2.0)^b^ | 28 d (14-49)^c^ | 0.325 (0.209-0.462)^b^ |  |
|  | Abdominal pain and distention (includes intestinal perforation) | 17.0 (10.0-25.7)^b^ |  | 0.324 (0.22-0.442)^b^ |  |
| Paratyphoid Fever | Mild | 28.5 (15.6-44.2)^b^ | 14 d (7-21)^c^ | 0.006 (0.002-0.012)^b^ |  |
|  | Moderate | 52.25 (27.2-77.7)^b^ |  | 0.051 (0.032-0.074)^b^ |  |
|  | Severe | 14.25 (8.2-21.8)^b^ | 28 d (14-49)^c^ | 0.133 (0.088-0.19)^b^ |  |
|  | Abdominal pain & distention due to paratyphoid | 5.0 (2.8-7.6)^b^ |  | 0.114 (0.078-0.159)^b^ |  |
| Toxoplasmosis | Moderate | - | 28 d | 0.051 | Lai and Yu (2020) |
| Cholera | Mild | 25 | 7 d | 0.074 |  |
|  | Moderate | 40 |  | 0.188 |  |
|  | Severe | 35 |  | 0.247 |  |
| Acute Hepatitis E | Moderate | 50 | 4 weeks | 0.051 (0.032-0.074)^b^ | Abbafati et al. (2020); GBD (2015) |
|  | Severe | 50 |  | 0.133 (0.088-0.19)^b^ |  |
| Listeriosis | - | - | 7 d | 0.210 (0.139-0.298)^b^ | de Noordhout et al. (2014) |

^a^ Mean (Range). ^b^ 95% CI. ^c^ 95% UI

**Appendix Table 6** Summary of data sources and DALY parameters for individual diseases notification categories - Direct-contact and Vector-borne transmitted diseases.

| **Notifiable infectious disease** | **Disease severity** | **Severity distribution (%)** | **Duration** | **Disability weights** | **Source** |
| --- | --- | --- | --- | --- | --- |
| **Direct-contact transmitted diseases** | | | | | |
| Leptospirosis | Chronic sequelae | 28 | 2 months-3 yr | 0.245 | Torgerson et al. (2015) |
|  | Mild | 33 | 2 months | 0.053 |  |
|  | Moderate | 26 |  | 0.21 |  |
|  | Severe | 7 | 2 weeks | 0.562 |  |
|  | Fatal | 1 | 1 month | 0.562 |  |
| Melioidosis | Moderate | 2.3 | 43.59 d | 0.051 (0.032-0.074)^b^ | Birnie et al. (2019) |
|  | Severe | 35.70 | 10.9 d | 0.133 (0.088-0.190)^b^ |  |
| Tetanus | - | - | 0.06-0.08 yr | 0.133 (0.088-0.19)^b^ | Abbafati et al. (2020); Cassini et al. (2018) |
| Hansen’s Disease | Disfigurement level 1 | 21.25 | 29.21 (22.53 SD) months | 0.011 (0.005-0.021)^b^ | Abbafati et al. (2020); Rathod et al. (2020); Masresha et al. (2020) |
|  | Disfigurement level 2 | 6.31 |  | 0.067 (0.044-0.100)^b^ |  |
| **Vector-borne transmitted diseases** | | | | | |
| Dengue Fever | Moderate | 94.5 | 6 d | 0.051 (0.032-0.074)^b^ | Abbafati et al. (2020) |
|  | Severe | 5.5 | 14 d | 0.133 (0.088-0.19)^b^ |  |
| Scrub Typhus | - | - | 14.4 d (9-19)^c^ | 0.197 | Taylor et al. (2015); Yang et al. (2015) |
| Japanese Encephalitis | Mild | 48 | 20 d (15.6-23.4)^a^ | 0.031 (0.018-0.05)^a^ | Vodicka et al. (2020) |
|  | Moderate | 24 (19-29)^a^ |  | 0.203 (0.134-0.29)^a^ |  |
|  | Severe | 28 (22-34)^a^ |  | 0.542 (0.374-0.702)^a^ |  |
| Malaria | Mild | 33.3 | 14-28 d | 0.006 (0.002-0.012)^b^ | Abbafati et al. (2020) |
|  | Moderate |  |  | 0.051 (0.032-0.074)^b^ |  |
|  | Severe |  |  | 0.133 (0.088-0.19)^b^ |  |
| Lyme Disease | - | - | 4.6 yr (3-6)^a^ | 0.364 (0.326-0.397)^b^ | van den Wijngaard et al. (2015) |
| Zika Virus Infection | Moderate | 41 | 6 d | 0.051 (0.032-0.074)^b^ | Abbafati et al. (2020); Gallian et al. (2017) |
|  | Asymptomatic | 59 |  | 0 (0-0) |  |

^a^ Mean (Range). ^b^ 95% CI. ^c^ Median (Range)

**Appendix Table 7** Summary of data sources and DALY parameters for individual diseases notification categories - Sexually and blood-borne transmitted diseases.

| **Notifiable infectious disease** | **Disease severity** | **Severity distribution (%)** | **Duration** | **Disability weights** | **Source** |
| --- | --- | --- | --- | --- | --- |
| Gonorrhea | - | - | 1 week | 0.006 (0.002-0.012)^b^ | Abbafati et al. (2020) |
| Syphilis | Primary | 50 | 5 yr | 0.007 (0.005–0.01)^b^ | Abbafati et al. (2020); Cassini et al. (2018) |
|  | Secondary | 4.5-7.5 |  | 0.125 (0.104-0.152)^b^ |  |
|  | Neurosyphilis | 0.75-1.88 |  | 0.407 (0.36-0.46)^b^ |  |
| Human immunodeficiency virus (HIV) infection/Acquired immunodeficiency syndrome (AIDS) | Symptomatic HIV | 4.5-7 | 5.36 yr | 0.274 (0.184-0.377)^b^ | Abbafati et al. (2020); Cassini et al. (2018) |
|  | AIDS with antiretroviral treatment | 70-90 | 8-10 yr | 0.078 (0.052-0.111)^b^ |  |
|  | AIDS without antiretroviral treatment | 32.09 | 0.08 yr | 0.582 (0.406-0.743)^b^ |  |
| Acute Hepatitis B/D | Moderate | 73 | 6 weeks | 0.051 (0.032-0.074)^b^ | Abbafati et al. (2020); GBD (2015) |
|  | Severe | 27 |  | 0.133 (0.088-0.19)^b^ |  |
| Acute Hepatitis C | Moderate | 24 | 6 weeks | 0.051 (0.032-0.074)^b^ |  |
|  | Severe | 1 |  | 0.133 (0.088-0.19)^b^ |  |

^a^ Mean (Range). ^b^ 95% CI
